# Classification of P-glycoprotein-interacting compounds using machine learning methods

**DOI:** 10.17179/excli2015-374

**Published:** 2015-08-19

**Authors:** Veda Prachayasittikul, Apilak Worachartcheewan, Watshara Shoombuatong, Virapong Prachayasittikul, Chanin Nantasenamat

**Affiliations:** 1 Center of Data Mining and Biomedical Informatics, Faculty of Medical Technology, Mahidol University, Bangkok 10700, Thailand; 2 Department of Clinical Microbiology and Applied Technology, Faculty of Medical Technology, Mahidol University, Bangkok 10700, Thailand; 3 Department of Clinical Chemistry, Faculty of Medical Technology, Mahidol University, Bangkok 10700, Thailand

**Keywords:** P-glycoprotein, ADMET, multidrug resistance, QSAR, data mining

## Abstract

P-glycoprotein (Pgp) is a drug transporter that plays important roles in multidrug resistance and drug pharmacokinetics. The inhibition of Pgp has become a notable strategy for combating multidrug-resistant cancers and improving therapeutic outcomes. However, the polyspecific nature of Pgp, together with inconsistent results in experimental assays, renders the determination of endpoints for Pgp-interacting compounds a great challenge. In this study, the classification of a large set of 2,477 Pgp-interacting compounds (i.e., 1341 inhibitors, 913 non-inhibitors, 197 substrates and 26 non-substrates) was performed using several machine learning methods (i.e., decision tree induction, artificial neural network modelling and support vector machine) as a function of their physicochemical properties. The models provided good predictive performance, producing MCC values in the range of 0.739-1 for internal cross-validation and 0.665-1 for external validation. The study provided simple and interpretable models for important properties that influence the activity of Pgp-interacting compounds, which are potentially beneficial for screening and rational design of Pgp inhibitors that are of clinical importance.

## Introduction

Human p-glycoprotein (Pgp) is a 170 kDa polypeptide (Juliano and Ling, 1976[24]) comprising 1280 amino acids (Chen et al., 1986[10]) and encoded by multidrug-resistance genes (Fardel et al., 2012[14]). Pgp is an ATP-binding cassette (ABC) transporter belonging to the ABCB subfamily (Hennessy and Spiers, 2007[21]) that functions as a dynamic efflux pump (Aller et al., 2009[2]) to transport substances out of cells (Hennessy and Spiers, 2007[21]). Notably, Pgp contains multiple binding sites that can non-specifically and simultaneously bind a wide range of structurally unrelated hydrophobic substances (Ambudkar et al., 2006[4]) including anticancer drugs (Bansal et al., 2009[7]). 

Pgp influences the pharmacokinetics of its substrate drugs due to its polyspecific binding nature and its expression in many physical barriers and pharmacokinetics-related organs (i.e., the gastro-intestinal (GI) tract, blood-brain-barrier (BBB), kidney, liver, endothelium and placenta) that function to limit the cellular uptake, distribution, excretion and toxicity of many substances and xenobiotics (Fardel et al., 2012[14]). The ability of Pgp to alter the pharmacokinetic profiles of its substrate drugs is considered to be a key factor that impairs treatment outcomes (Krishna and Mayer, 2000[26]). In addition, the identification of Pgp substrates is essential for early ADMET screening, as recommended by FDA guidelines (U.S. Food and Drug Administration, 2012[44]). Drug-drug interactions and undesirable side effects are also important when drugs with narrow therapeutic windows are co-administered with strong Pgp inhibitors (Amin, 2013[5]; Aszalos, 2007[6]; Wessler et al., 2013[47]). 

Pgp is considered to be a lucrative target against multidrug-resistant cancers (Juliano and Ling, 1976[24]). Pgp over-expression is found in many types of cancer and its association with multidrug-resistance mechanisms has been attributed to impaired delivery of anticancer drugs to target cells (Hennessy and Spiers, 2007[21]). Therefore, the inhibition of Pgp has been considered to be an effective strategy for improving the therapeutic outcome of affected Pgp substrates, as well as combating multidrug resistance (Szakács et al., 2006[42]). 

The promiscuity of Pgp is an important issue that renders the classification of its interacting compounds a great challenge. Many experimental assays using multiple measurements and criteria are available for determining the end-points of Pgp-interacting compounds as substrates, non-substrates, inhibitors and non-inhibitors (Heredi-Szabo et al., 2013[22]; Li, 2005[28]; Polli et al., 2001[36]). The discordance of experimental assays has led to conflicting reports of their end-points (Seelig, 1998[38]; Sharom, 1997[39]). In addition, Pgp is a highly flexible protein containing multiple binding sites with different affini-ties for distinct compounds (Zeino et al., 2014[50]). Therefore, the classification of Pgp compounds is not an easy task because of the promiscuity of this transporter (Wang et al., 2005[45]). For these reasons, computational approaches have become versatile tools for exploring protein-ligand interactions (Nantasenamat et al., 2009[30]; Nantasenamat et al., 2010[31]; Nantasenamat and Prachayasittikul, 2015[32]) and is thus crucial for understanding Pgp-ligand interaction. Recently, quantitative structure-activity relationship (QSAR) studies (Ghandadi et al., 2014[16]; Palestro et al., 2014[34]; Shen et al., 2014[40]), classification models (Adenot and Lahana, 2004[1]; Chen et al., 2011[12]; Klepsch et al., 2014[25]; Levatić et al., 2013[27]; Li et al., 2014[29]; Penzotti et al., 2002[35]; Wang et al., 2011[46]), molecular docking (Ghandadi et al., 2014[16]; Palestro et al., 2014[34]; Zeino et al., 2014[50]) and homology modelling approaches (Yamaguchi et al., 2012[49]) have been used in an attempt to address these controversial issues. It is known that Pgp is one of the most studied drug transporters (Gottesman et al., 2002[18], 1996[19]). Despite extensive studies, the classification rules for interacting ligands are still not fully understood (Chen et al., 2012[11]; Levatić et al., 2013[27]).

**Figure 1:**
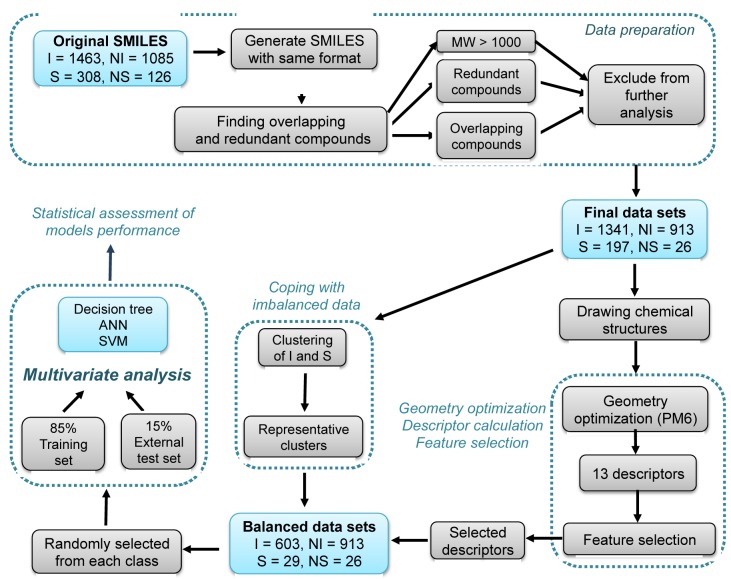
Schematic workflow of this study consisted of 4 major steps: (1) data sets preparation, (2) determining informative molecular descriptors, (3) coping with imbalanced data and (4) multivariate analysis. In step 1, redundant compounds, overlapping compounds, and compounds with MW > 1000 Da were identified and removed. Next, in step 2 the resulting compounds from the aforementioned pre-processed data sets were geometrically optimized at the PM6 level, calculate a set of 13 descriptors, apply feature selection to select informative descriptors for multivariate analysis. Subsequently, in step 3 the imbalanced number of positive and negative classes solved by making sure that positive class clusters were equivalent in number to that of the negative class where clusters providing the best predictive performance are selected as the representative clusters for model construction. Finally, in step 4 the balanced data set was subjected to data splitting via random selection into a training (85 %) and external test (15 %) set. Predictive models were constructed using DT, ANN and SVM algorithms. Predictive performance of the models were assessed by a set of statistical parameters. I = inhibitors, NI = non-inhibitors, S = substrates, NS = non-substrates.

Machine learning techniques are computational methods that have been successfully used for constructing predictive models and classifiers of Pgp-interacting compounds (Broccatelli, 2012[8]; Gombar et al., 2004[17]; Klepsch et al., 2014[25]; Li et al., 2014[29]). In this study, several machine learning classifiers were used to classify a large set of 2,477 compounds (i.e., 1341 inhibitors, 913 non-inhibitors, 197 substrates and 26 non-substrates) as a function of their physicochemical properties (Figure 1(Fig. 1)). This study provides a glimpse of the underlying classification criteria for Pgp-interacting compounds, which are potentially beneficial for the screening and design of Pgp inhibitors for clinical applications.

## Materials and Methods

### Data set

A data set of Pgp-interacting compounds was retrieved from the admetSAR database created by Cheng et al. (2012[11]). The database is a compilation of chemical structures gathered from different literature sources. It is represented in simplified molecular input line entry system (SMILES) format together with the Pgp classification labels (i.e., inhibitors, non-inhibitors, substrates and non-substrates). Owing to the inherent multiplicity and heterogeneity presented in SMILES notation, it was necessary to convert them to a uniform representation using the command line version of MarvinSketch, version 6.3.1 (ChemAxon, 2014[9]). Consequently, all newly generated SMILES, along with their Pgp class label, were combined within a single Excel worksheet. Compounds with molecular weight greater than 1000 Da and redundant compounds were identified and removed from further analysis. In addition, the compounds that were classified as belonging to more than one class were defined as overlapping compounds and were discarded from the analysis. This resulted in a final data set containing 1341 inhibitors, 913 non-inhibitors, 197 substrates and 26 non-substrates. A schematic workflow of the data set preparation is shown in Supplementary Figure S1.

### Geometry optimization and descriptor calculation

SMILES of all compounds were converted to .mol files and further processed for suitable formatting using in-house developed scripts. All chemical structures were geometrically optimized using Gaussian 09 at the semi-empirical level using the parameterization method 6 (PM6) approach (Frisch et al., 2009[15]). The optimized structures were used for extraction and calculation of the molecular descriptors. Initially, a set of 13 simply interpreted descriptors, including 6 quantum chemical descriptors and 7 molecular descriptors, was selected to represent the physicochemical properties of the compounds. A set of 6 quantum chemical descriptors was calculated and extracted from the optimized chemical structures. The six quantum chemical descriptors included the mean absolute charge (*Q*_m_), energy, dipole moment (*µ*), highest occupied molecular orbital (HOMO), lowest unoccupied molecular orbital (LUMO) and the energy of the HOMO and LUMO gap (HOMO-LUMO). The optimized structures were further used as input files for the calculation of an additional 7 molecular descriptors using Dragon 5.5 Professional (Talete, 2007[43]). The seven molecular descriptors include molecular weight (MW), rotatable bond number (RBN), number of rings (nCIC), number of hydrogen bond donors (nHDon), number of hydrogen bond acceptors (nHAcc), Ghose-Crippenoctanol-water partition coefﬁcient (ALogP), and the topological polar surface area (TPSA). 

### Feature selection

Feature selection was performed on the initial set of 13 descriptors using the SPSS version 18 software (Inc.) (IBM, SPSS Inc., USA[23]). The inhibitor and non-inhibitor classes, along with their 13 descriptor values, were combined. The intercorrelation matrix of Pearson's correlation coefficients was calculated and a cut-off value of 0.7 was used for identifying collinear and redundant descriptors. For any given pair of descriptors whose correlation coefficient values were ≥ 0.7, one of them was discarded. The same procedure was carried out on the combined data of substrates and non-substrates. Finally, the resulting set of descriptors was subsequently used for multivariate analysis.

### Solving the imbalanced data set issue

The fuzzy C-means clustering (FCM) algorithm is an unsupervised machine learning algorithm that is widely used for clustering, feature analysis and classifier design (Zhou et al., 2010[51]). It is a clustering algorithm that confirms to what degree the samples belong to a certain class (Zhou et al., 2010[51]). The principle of FCM is provided in the supplementary information. In this study, the FCM algorithm was used to select representative samples from the positive class (i.e., inhibitors and substrates) using the R software environment (R Development Core Team, 2010[37]). Firstly, clusters were generated from the ratio of the number of samples in the positive class to the number of samples in the negative class (non-inhibitors and non-substrates). Second, the decision tree model was constructed using the generated clusters from the positive class data sets together with the negative class data set (Witten et al., 2011[48]). A combination of statistical parameters comp of accuracy, sensitivity, specificity and Matthews' correlation coefficient (MCC) were used for determining the best clusters. Finally, the positive class clusters showing the best predictive performance were selected as the representative data set for further multivariate analysis (Table S1).

### Model development

**Figure 2:**
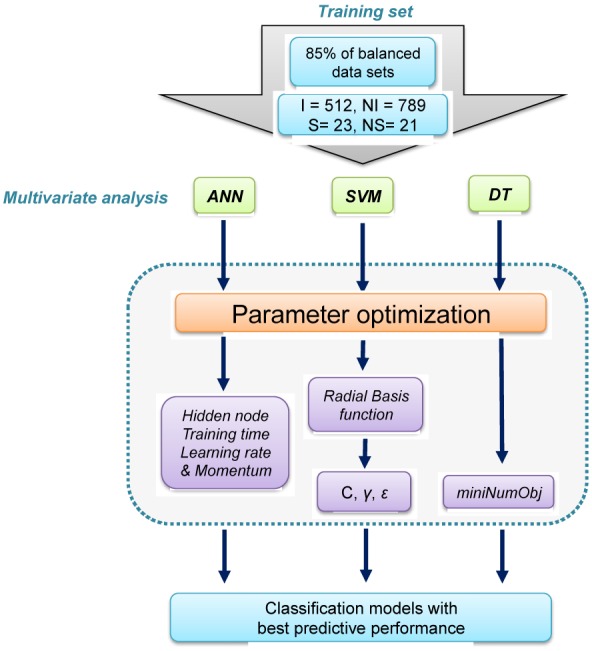
Workflow of the parameter optimization process of learning classifiers (e.g. ANN, SVM and DT). Parameter optimization was performed as to search for the optimal value of learning parameters that will afford the best predictive performance. Identified optimal parameters were then employed in construction of the final model.

The classification structure-property relationship (CSPR) models were used for revealing the relationships between the descriptor values and the classification of Pgp-interacting compounds. A random sampling method was used for dividing each data set into two separate groups containing 85 % and 15 % of the whole data, respectively. For each class (i.e., selected inhibitors cluster, non-inhibitors, selected substrates cluster and non-substrates), the data subset containing 85 % of the compounds was used in the construction of predictive models (constitutes the internal validation). However, the second data subset containing 15 % of the compounds was used for external validation. Random sampling was performed by means of principal component analysis (PCA) using the R software environment (R Development Core Team, 2010[37]). Finally, 85 % of the selected positive class clusters (512 inhibitors and 23 substrates) were used, together with 85 % of the negative class clusters (789 non-inhibitors and 21 non-substrates) for the construction of CSPR models (Figure 1(Fig. 1)). Three classifiers were employed for prediction, namely decision tree (DT), artificial neural network (ANN) and support vector machine (SVM). The former was calculated using the Weka software package version 3.7.11 (Witten et al., 2011[48]). The latter two classifiers were calculated using an in-house automated data mining software program called AutoWeka (Nantasenamat et al., 2015[33]), which was implemented as a Python wrapper built on top of Weka. The procedures for parameter optimization of each algorithm are illustrated in Figure 2(Fig. 2). Information on the principles and parameter optimization methods for each classifier are provided in the Supplementary Information and Supplementary Tables S2-S4.

### Validation of predictive models

The *k*-fold cross validation (*k*-fold CV) method is widely accepted for the measurement of predictive performance of classification models (Ambroise and McLachlan, 2002[3]; Hastie et al., 2001[20]; Subramanian and Simon, 2011[41]). Briefly, a data set of *n* samples is randomly divided into *k* subsets. Subsequently, *k*-1 subsets are used as the training set, whereas 1 subset is used as the test set. This process continues until every subset is used as the test set. In this study, 10-fold CV was used for internal validation of the constructed models.

In addition to internal validation of the predictive models, external validation using external test sets was performed. As mentioned, 85 % of the compounds in each class are randomly selected for the construction of the models and internal validation.

The remaining subset containing 15 % of the compounds were subsequently used for external validation. Therefore, additional models were constructed by using the 85 % subset for each class as the training set while applying the resulting model on the 15 % subset that serve as the external test set (Figure 1(Fig. 1)). 

### Statistical assessment of the predictive models

The predictive performance of the CSPR models was assessed using a combination of statistical parameters (i.e., accuracy, sensitivity, specificity and MCC) to interrogate all aspects of the models, as shown in Equations [1]-[4]
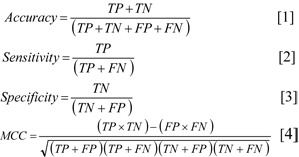
. 

where TP is the number of true positives, TN is the number of true negatives, FP is the number of false positives or over-predictions and FN is the number of false negatives or missed predictions. 

The accuracy is used for determining the degree of correct predictions relative to the total number of samples. The sensitivity is a true positive rate that represents the actual positives that are correctly classified. The specificity is a true negative rate that determines the actual negatives that are correctly classified. Accuracy, sensitivity and specificity were calculated as percentages. However, these parameters may not provide a comprehensive analysis of the models. Therefore, a balanced statistical parameter method, Matthews correlation coefficient (MCC), was additionally used. The MCC is calculated using both true and false positives and negatives. MCC is used as a balanced measurement for binary classification, and it can be used with imbalanced data containing different sizes of classes. 

## Results and Discussion

### Feature selection

Redundant descriptors were identified and removed using a cut-off value of 0.7. The intercorrelation matrix for both models is displayed in Supplementary Figure S2. For the inhibitors/non-inhibitors set, 2 redundant descriptors (i.e., MW and TPSA) were removed and the remaining 11 descriptors were used for the construction of the CSPR models. Similarly, 2 redundant descriptors (i.e., nHAcc and Energy) were removed from the substrates/non-substrates set, which resulted in a set of 11 descriptors for subsequent CSPR model building. 

### Coping with imbalanced data sets

The data sets for the positive class compounds (i.e., 1341 inhibitors and 197 substrates) were clearly imbalanced relative to those of the negative class compounds (i.e., 931 non-inhibitors and 26 non-substrates). Therefore, FCM was used to select representative samples from the positive class (i.e., inhibitors or substrates). The results of the predictive performance of classification models constructed from the original data sets of positive class compounds and their clusters are provided in Table S1. The representative clusters of positive class compounds were selected with respect to their best predictive performance for multivariate analysis (i.e., 603 inhibitors and 27 substrates). CSPR models of inhibitors/non-inhibitors and substrates/non-substrates were separately constructed using DT, ANN and SVM analysis. For each class, a random sampling was performed by principal components analysis (PCA) using the R software environment (R Development Core Team, 2010[37]) to create a training set (85 %) and an external test set (15 %), as summarized in Figure 1(Fig. 1).

### Multivariate analysis using DT, ANN and SVM

**Figure 3:**
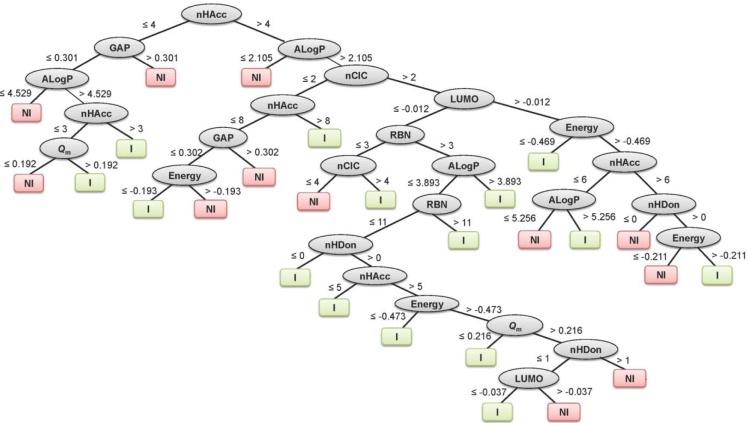
Decision tree for classifying inhibitors (I) and non-inhibitors (NI)

**Figure 4:**
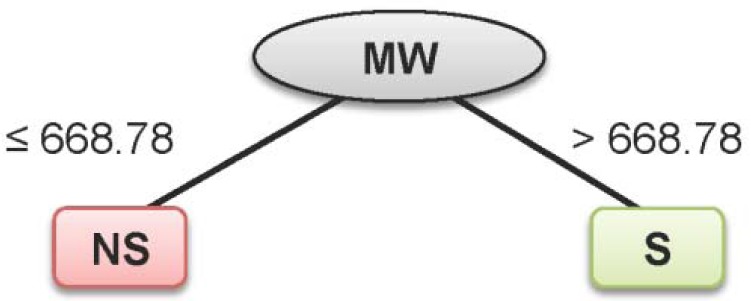
Decision tree for classifying substrates (S) and non-substrates (NS)

Summaries of the true positive (TP), false positive (FP), false negative (FN) and true negative (TN) values for each classifier are provided in Table 1(Tab. 1). Summaries of the predictive performance of the DT, ANN and SVM models of inhibitors/non-inhibitors and substrates/non-substrates are shown in Tables 2(Tab. 2) and 3(Tab. 3), respectively. A series of if-then rules for classifying compounds was obtained from decision trees of inhibitors/non-inhibitors and substrates/non-substrates, as displayed in Figures 3(Fig. 3) and 4(Fig. 4), respectively.

The inhibitors/non-inhibitors model provided greater than 85 % accuracy, sensitivity and specificity for all investigated data sets, except for the sensitivity of the external test set (Sens_ext_ = 79.121 %). In addition, the MCC values showed that the models are capable of classifying both negative and positive classes, as evidenced by MCC values of 0.832, 0.739 and 0.743 for training, 10-fold CV and external validation, respectively.

The decision tree indicated that 8 descriptors were selected for splitting data (i.e., nHAcc, HOMO-LUMO gap, ALogP, *Q*_m_, Energy, nCIC, RBN and LUMO). The nHAcc was selected as the root node with a cut-off criterion of 4 (Figure 3(Fig. 3)). Likewise, good performance was obtained for the substrates/non-substrates model affording high accuracy, ranging from 88.89 to 100 %.

Similar results were found for specificity, with values ranging from 80 to 100 %. As for the sensitivity, 100 % accuracy was obtained for all investigated data sets. In addition, the model provided high performance for the prediction of both classes, as determined by the high MCC values (i.e., MCC_tr_ = 1, MCC_cv_= 0.955 and MCC_ext_= 0.800). Notably, only MW was selected from the set of 11 descriptors in the construction of a single node decision tree for its classification with a cut-off value of 668.78 (Figure 4(Fig. 4)).

The inhibitors/non-inhibitors ANN model provided high predictive performance, as indicated by MCC values of 0.759 and 0.703 for 10-fold CV and the external test set, respectively. The training set provided values greater than 90 % for accuracy, sensitivity and specificity, whereas values greater than 87 % and 80 % for these three parameters were obtained for the 10-fold CV and external test set, respectively. Notably, the substrates/non-substrates ANN model provided remarkable prediction as evidenced by the 100 % accuracy, sensitivity and specificity, as well as the MCC score of 1, which was the highest possible score.

In summary, the three classifiers (e.g. DT, ANN and SVM) provided good predictive classification models, as deduced from their high statistical parameters. For the classification of inhibitors/non-inhibitors, the highest MCC value (0.759) and accuracy (88.378) of 10-fold CV were afforded by the ANN model, whereas the best predictive performance of the external test set was provided by decision tree analysis, which produced an MCC = 0.743 and accuracy = 87.442 %.

As for the classification of substrates/ non-substrates, ANN and SVM afforded the same prediction performance level, producing correctly classified instances 100 % of the time in training, 10-fold CV and external validation. The decision tree model showed acceptable prediction with an MCC of 0.955 and 0.800 for 10-fold CV and the external test set, respectively. However, it was found from the external validation that one non-substrate was incorrectly classified as a substrate. The chemical structure of this compound (Supplementary Figure S3) and its calculated descriptor values are provided in the Supplementary Information.

From the decision tree model of the substrates/non-substrates data set (Figure 4(Fig. 4)) it can be seen that MW was the single and most important feature for classification with a cut-off value of 668.78, and any compounds with their MW greater than this cut-off value were classified as substrates. The MW of this incorrectly classified compound is 692.80; therefore, it was misclassified as a substrate. 

## Conclusions

The promiscuity of Pgp renders the determination of its ligand endpoints a great challenge. In this study, three classifiers (e.g. DT, ANN and SVM) were used to classify 2,477 compounds as Pgp-interacting or non-interacting, as a function of eleven important descriptors. The predictive model provided insights into important physicochemical properties governing the activity of compounds towards the Pgp transporter, as well as suggesting pertinent classification criteria that could be beneficial for the screening and design of Pgp inhibitors for a wide range of therapeutic applications. 

## Notes

Supplementary information is available on the EXCLI Journal website.

## Conflict of interest

The authors have declared that no competing interests exist.

## Acknowledgements

We gratefully acknowledge financial support from the following agencies: the Mahidol University Postdoctoral Fellowship Program (to W.S. under the supervision of V.P. and C.N.), the Mahidol University Talent Management Program (to A.W.), the Mahidol University Goal-Oriented Research Grant (to C.N.) and the Office of the Higher Education Commission and Mahidol University under the National Research Universities Initiative.

## Supplementary Material

Supplementary

## Figures and Tables

**Table 1:**
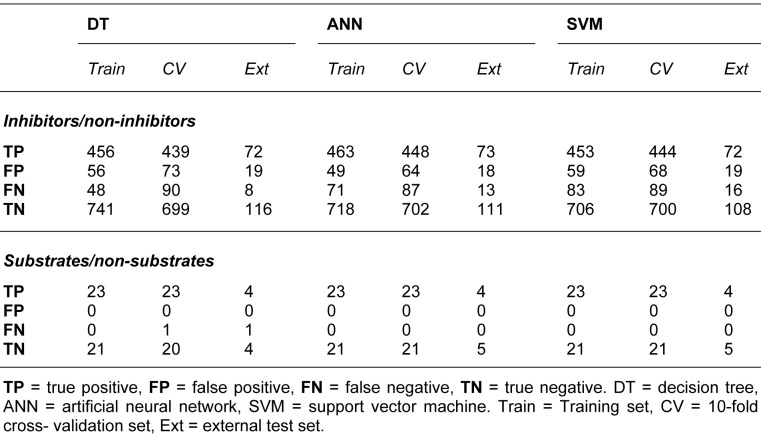
Summary of true and false positives/negatives of three classifiers

**Table 2:**
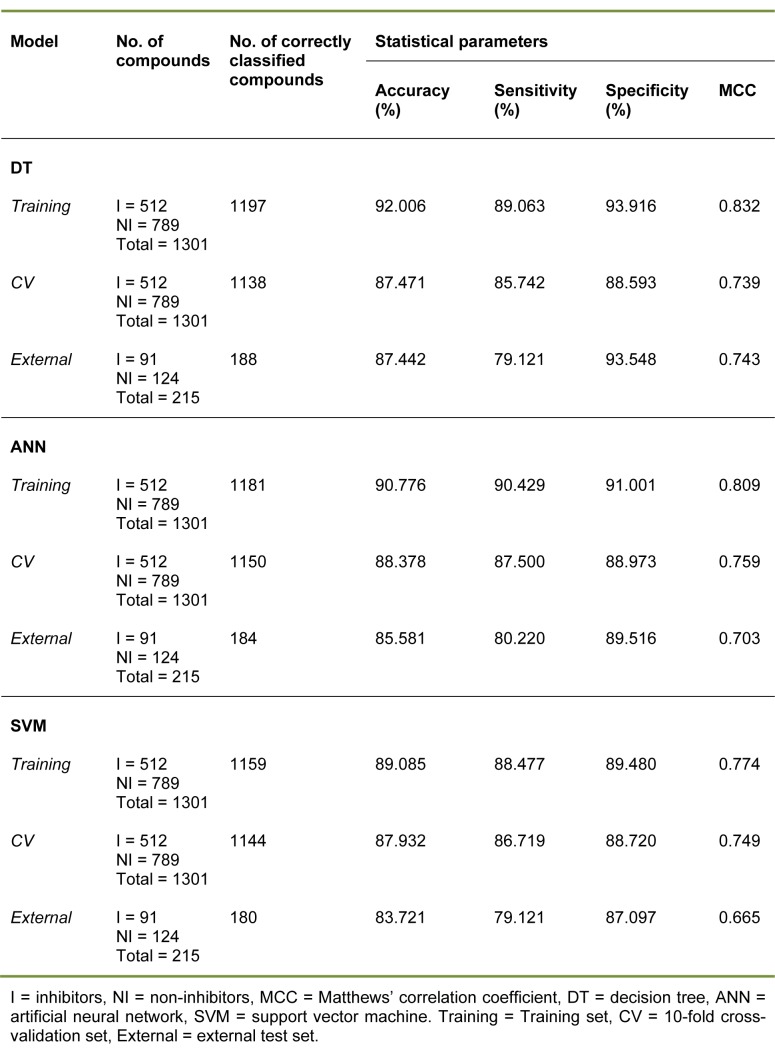
Summary of predictive performance for classifying inhibitors and non-inhibitors using several machine learning classifiers

**Table 3:**
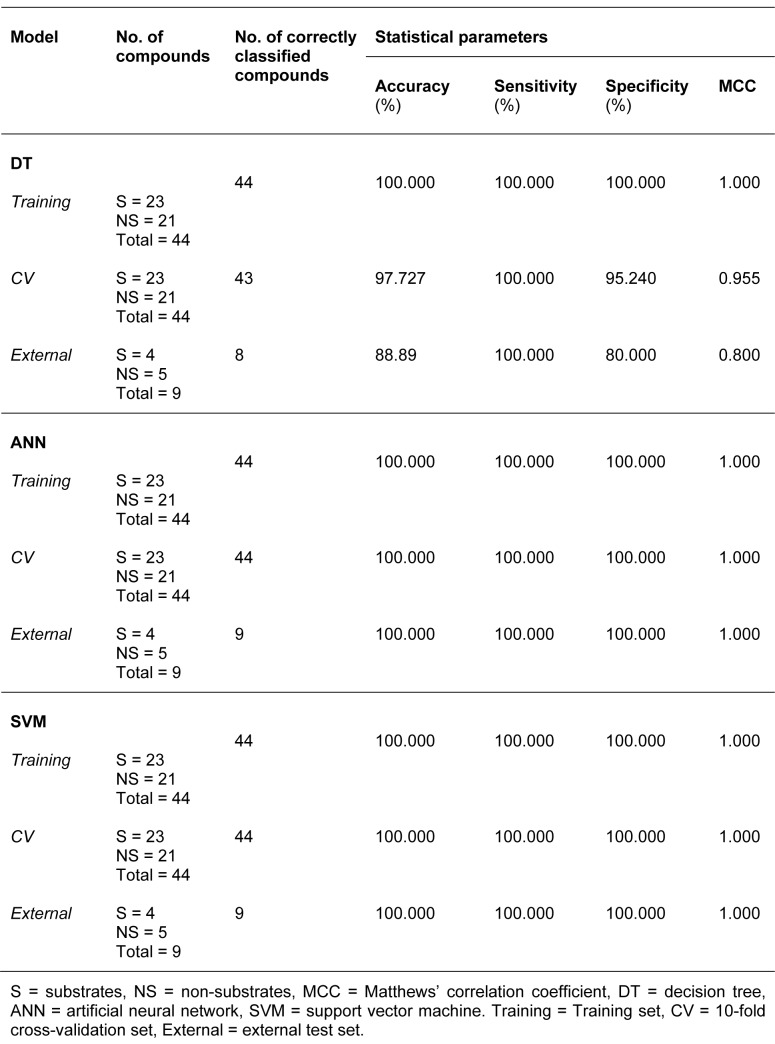
Summary of predictive performance for classifying substrates and non-substrates using several machine learning classifiers
