# Supplementary material for: Classification of P-glycoprotein-interacting compounds using machine learning methods
Source: EXCLI J. 2015 Aug 19;14:958–70. doi: 10.17179/excli2015-374 (PMC4743480; doi:10.17179/excli2015-374)
Supplement: Supplementary [file EXCLI-14-958-s-001.pdf]

EXCLI Journal 2015;14:958-970 – ISSN 1611-2156
Received: May 27, 2015, accepted: July 11, 2015, published: August 19, 2015

Supplementary material to:

CLASSIFICATION OF P-GLYCOPROTEIN-INTERACTING COMPOUNDS USING MACHINE LEARNING METHODS

Veda Prachayasittikul[1, 2], Apilak Worachartcheewan[1, 3], Watshara Shoombuatong[1], Virapong Prachayasittikul[2], Chanin Nantasenamat[1, 2,*]

[1] Center of Data Mining and Biomedical Informatics, Faculty of Medical Technology, Mahidol University, Bangkok 10700, Thailand
[2] Department of Clinical Microbiology and Applied Technology, Faculty of Medical Technology, Mahidol University, Bangkok 10700, Thailand
[3] Department of Clinical Chemistry, Faculty of Medical Technology, Mahidol University, Bangkok 10700, Thailand

* Corresponding author: E-mail: chanin.nan@mahidol.ac.th (C.N.); Phone: +66 2 441 4371; Fax: +66 2 441 4380

http://dx.doi.org/10.17179/excli2015-374

This is an Open Access article distributed under the terms of the Creative Commons Attribution License (http://creativecommons.org/licenses/by/4.0/).

Supplementary Information

Data set

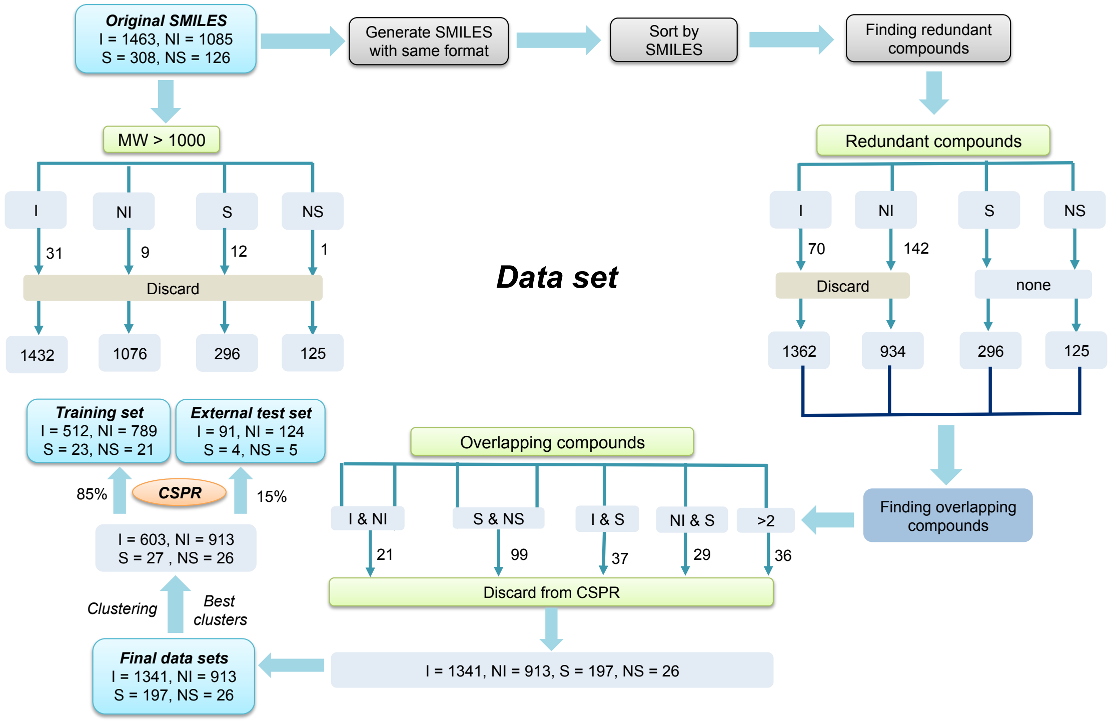

Fig. S1. A schematic workflow of the data set preparation.

Feature selection

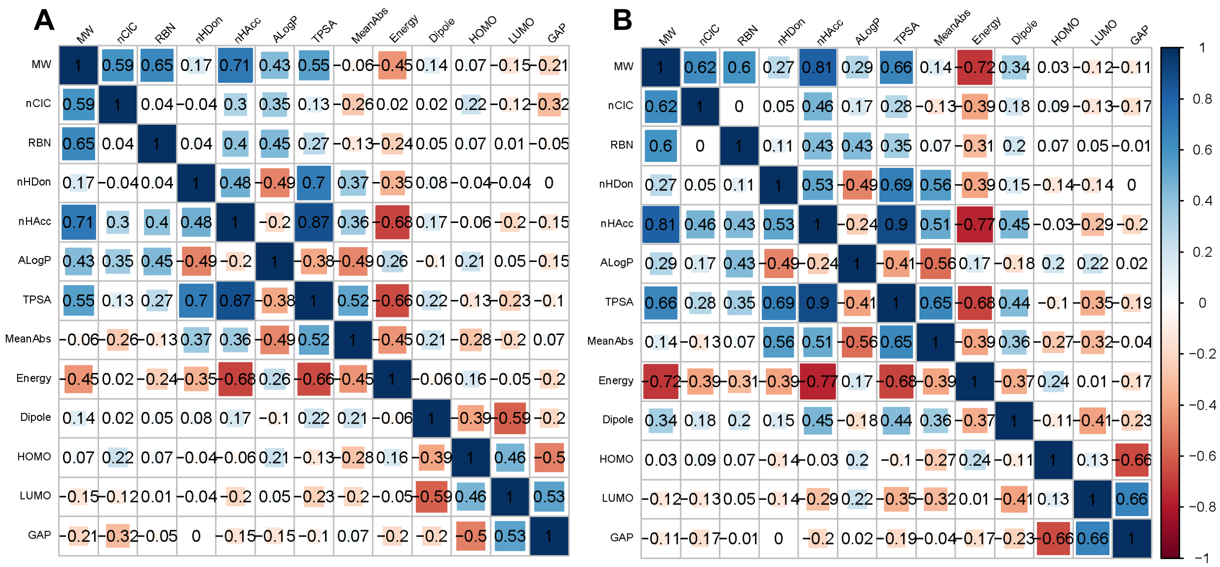

Fig. S2. The intercorrelation matrix (using Pearson's coefficient values) of the inhibitors/non-inhibitors model (A) and substrates/non-substrates model (B).

Coping with imbalanced data sets

The fuzzy C-means clustering (FCM) algorithm divides the input data into many clusters in which every data point possesses a partial membership in multiple clusters rather than complete association with a single cluster. Therefore, data points in the center of a cluster have a greater degree of belonging than data points located at the edge of cluster. Initially, FCM divides the *n* vector of x_i (i = 1, 2, 3,…, *N*) into *c* fuzzy groups, where $x_i \in \Re^M$. The clustering center of each group is subsequently calculated, and the non-similarity index value function is minimized. For determination of membership in a cluster, a value of 0 or 1 is given to each data point. Subsequently, the element of the membership matrix is provided with the values of 0 and 1. The optimal cluster of each x_i was obtained by minimizing the objective function of FCM (Zhou et al., 2010):

$$J_m = \sum_{i=1}^{c} \sum_{j=1}^{n} u_{ij}^{m} \left\| x_i - c_i \right\|^2 \qquad (1)$$

where *m* is a real-valued number greater than 1, u_{ij}^{m} is the degree of membership of x_i in the cluster *j*, c_j is the center of the cluster *j*, and $\|\ \|$ is the similarity function between any measured data and the center. In this study, the number of clusters was generated from the ratio of the number of samples in the positive class to the number of samples in the negative class. The ratio of inhibitors:non-inhibitors was approximately 1.47; therefore, 2 clusters were generated for the inhibitors data set. These clusters were consequently used to represent the inhibitors class, along with all 913 inhibitors, for construction of the classification models. Herein, the decision tree algorithm was used for empirical observation. The predictive performance of each model was compared using a set of statistical parameters, including % accuracy (Acc), % sensitivity (Sens), % specificity (Spec) and Matthews correlation coefficient (MCC). Finally, inhibitors cluster 2 was selected as the best representative of the inhibitors for further CSPR analysis.

Table S1. A summary of predictive performance of positive class clusters

Class	Original / Cluster	No. of I + NI / S + NS	ACC_{tr} (%)	$^{a}ACC_{cv}$ (%)	$Sens_{tr}$ (%)	$^{a}Sens_{cv}$ (%)	$Spec_{tr}$ (%)	$^{a}Spec_{cv}$ (%)	MCC_{tr}	$^{a}MCC_{cv}$
I	Original	1341 + 913	89.219	82.165	91.723	87.696	85.542	74.042	0.776	0.627
I	Cluster 1	738 + 913	91.217	84.858	89.431	84.688	92.662	84.995	0.822	0.695
I	Cluster 2[b]	603 + 913	92.414	86.675	90.879	82.919	93.428	89.157	0.842	0.722
S	Original	197 +26	96.413	85.202	99.492	93.909	73.077	19.231	0.815	0.159
S	Cluster 1	14 + 26	97.500	85.000	100.000	78.571	96.154	88.462	0.947	0.670
S	Cluster 2	26 + 26	96.154	90.385	100.000	96.154	92.308	84.615	0.926	0.813
S	Cluster 3[b]	27 + 26	100.000	98.113	100.000	100.000	100.000	96.154	1.000	0.963
S	Cluster 4	21 + 26	91.489	87.234	100.000	85.714	84.615	88.462	0.843	0.742
S	Cluster 5	36 + 26	96.774	77.420	100.000	83.333	92.310	69.231	0.940	0.530
S	Cluster 6	27 + 26	98.113	92.453	100.000	96.296	96.154	88.462	0.963	0.851
S	Cluster 7	46 + 26	90.277	73.611	97.826	78.261	76.923	65.385	0.789	0.433

I = inhibitor, NI = non-inhibitor, S= substrate, NS = non-substrate, ACC = accuracy, Sens = sensitivity, Spec = specificity, MCC = Matthews correlation coefficient.

[a] 10-fold cross validation was performed for internal validation of the models.

[b] The cluster that gives the best MCC was selected as representative for further CSPR analysis.

Multivariate analysis

Decision tree

Decision tree analysis is a supervised machine-learning algorithm (Tarca et al., 2007; Witten et al., 2011) that has been widely used as a simple interpretation of binary classification (Tarca et al., 2007). Decision tree analysis is a way to represent a series of rules that leads to a particular classification (Sharma & Jain, 2013). It divides input data into a range based on attribute values that it learned from the training data set (Patil & Sherekar, 2013). In this study, decision tree models were constructed using the J48 algorithm of the Weka software package version 3.7.11. (Witten et al., 2011). The process of J48 starts with creating if-then rules from the whole training set to split the data into two subsets, in which each subset contains data with the same feature value (Che et al., 2011). The splitting is performed through the use of internal nodes (i.e., independent variables) and external nodes (i.e., dependent variables) connected by branches (i.e., the cutoff value determining the class of the compounds) (Nantasenamat et al., 2013a). The tree initially finds and selects the most informative attribute (i.e., a descriptor as a root node for splitting data), followed by subsequent important attributes as internal nodes, until the terminal branch is reached (Nantasenamat et al., 2013a). The process continues until all samples in a subset are of the same class (Che et al., 2011). Initially, a large tree is grown and then pruned to reduce overfitting (Che et al., 2011). To produce a simple interpreted tree with the best performance, the minimum number of instances per leaf (miniNumObj) was optimized. The models were empirically constructed using varied miniNumObj. In addition, the validation set was used for an empirical search of suitable parameters. Matthews correlation coefficient (MCC) values for the training set (MCC_{tr}), 10-fold cross validation (MCC_{cv}) and a validation set (MCC_{v}) were used to determine the predictive performance of the model. Finally, the miniNumObj that gave the best MCC value was further used for the construction of the CSPR model based on J48.

The results of classification using varied miniNumObj of the inhibitors/non-inhibitors classifier are shown in Table S2. The best predictive performance of the inhibitors/non-inhibitors model was provided by the miniNumObj of 8. Regarding the predictive performance, the results of the models using this miniNumObj parameter were selected as final.

Table S2. The parameter optimization of the decision tree models

Model	miniNumObj	No. of leaves	Size of tree	[a]MCC_{tr}	[b]MCC_{cv}	[c]MCC_{ext}
I_NI	2	40	79	0.877	0.708	0.694
I_NI	3	32	63	0.857	0.724	0.703
I_NI	4	31	61	0.852	0.715	0.703
I_NI	5	34	67	0.860	0.709	0.713
I_NI	6	31	61	0.860	0.733	0.723
I_NI	7	29	57	0.850	0.739	0.732
I_NI	8[d]	27	53	0.832[d]	0.739[d]	0.743[d]
I_NI	9	13	37	0.812	0.742	0.694
I_NI	10	14	27	0.792	0.739	0.674
S_NS	2[d]	2	3	1.000[d]	0.955[d]	0.800[d]
S_NS	3	2	3	1.000	0.955	0.800
S_NS	4	2	3	1.000	0.955	0.800

[a]Training. [b]10-fold cross validation. [c] External test set. [d] The best predictive performance.

Incorrectly classified compound

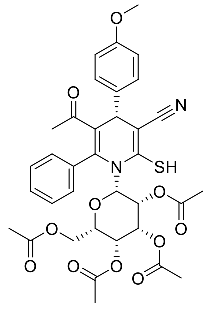

Fig. S3. An incorrectly classified compound from the decision tree substrates/non-substrates model.

An incorrectly classified compound from the substrate/non-substrate decision tree classifier is shown in Fig. S3. The calculated descriptor values of this compound are MW = 692.8, nCIC = 4, RBN = 14, nHDon = 0, ALogP =3.273, TPSA = 206.56, *Q*m = 0.266894306, Dipole = 13.0767, HOMO = -0.32477, LUMO = -0.03158 and HOMO-LUMO GAP = 0.29319.

Artificial neural network (ANN)

ANN is a supervised learning algorithm that mimics the behavior of the human brain, where the neuronal nodes of ANN represent human neurons and the synaptic weights represent dendrites and axons (Nantasenamat et al., 2013b). ANN is comprises many artificial processing units located in 3 layers, including input, hidden and output layers (Sutariya et al., 2013). The strength of the connection between processing units is defined by synaptic weights that can be adjusted by the learning process (Sutariya et al., 2013). The values of independent variables are relayed to the input layers, and then the signals are sent to hidden layers and output layers via synaptic weights (Nantasenamat et al., 2013b). The artificial neurons in the hidden layers contain a sigmoidal transfer function $\mathrm{S}(x)$ (Eq. 2), which computes and limits the signal of the output layer as 0 or 1 (Nantasenamat et al., 2013b).

$$\mathrm{S}(x) = \frac{1}{1 + e^{-\beta x}} \qquad (2)$$

where β is the slope parameter. The output layer contains a numerical class that is an unthresholded linear unit. In mathematical models, it may describe a neuron *k*, as follow:

$$\hat{y} = S\left(\sum_{i=1}^{N} w_{ki} x_i - w_0\right) \qquad (3)$$

where $w_{ki} = w_{k1}, w_{k2}, \ldots, w_{kM}$ is the weight of neuron which is optimized by total squared error, w_o. is the weight which corresponds to the bias input, and $\hat{y}$ is the output signal of the neuron.

The model is trained in a back-propagated manner, in which the difference between $\hat{y}$ and y is calculated as the target error from the output layer through the hidden layer to the input layer, followed by a readjustment of the synaptic weights (Nantasenamat et al., 2013b). This process continues until reaching the assigned learning period and obtaining a minimized error and good prediction (Sutariya et al., 2013). The initial synaptic weights are randomly assigned at the beginning of the learning process, which may give rise to a slight varied prediction. Therefore, ten rounds of calculations were performed and the average parameter value was calculated and used for construction of the ANN models.

The optimal value of parameters for ANN, including the number of hidden nodes, training time and learning rate and momentum, were empirically searched by software developed in-house, i.e., Autoweka. Ten calculations were performed, and the average RMSE values from these rounds were used to measure the predictive performance (Nantasenamat et al., 2013b). The optimal parameters are shown in Table S3.

Table S3. Optimal parameters of the ANN models

Model	Hidden node	Training time	Learning rate	Momentum	$RMSE_{tr}$	$RMSE_{cv}$
I_NI	17	200	0.3	0.0	0.2659	0.2934
S_NS	1	1000	0.8	0.6	0.0044	0.0172

Support vector machine (SVM)

SVM is a supervised machine-learning algorithm based on statistical learning theory (Vapnik, 1998; Vapnik, 2000). SVM is a classifier that can separate data from two classes by finding a unique separating hyperplane with maximum margin (Vapnik, 2000) to minimize the classification error (Zhou et al., 2010). SVM searches for a set of data points that are the most difficult training points to be classified, which are defined as support vectors (Vapnik, 2000). These support vectors are closest to the hyperplane and located on the margin boundaries between the two classes (Yang, 2004). These striking characteristics contribute to the robustness and generalization ability of this classifier (Yang, 2004). Non-linear SVM was used in this study. Initially, non-linear original input X is projected into a higher dimensional feature space to allow the non-linear original data to be linearly separated in the transformed space using the kernel function (Eq.4) (Tarca et al., 2007).

$$K(x_i, x_j) = (\varphi(x_i) \cdot \varphi(x_j)) \tag{4}$$

where *K()* represents the kernel function and φ is a mapping function from the original input space into the feature space. Consequently, the linear classification model was constructed in the higher dimensional feature space, where the similarity between input data and support vectors was quantified by the kernel function, i.e., the radial basis function (RBF), as shown in Eq.5.

$$K(x_i, x_j) = \exp(-\gamma \|x_i - x_j\|) \tag{5}$$

where *K()* represents the kernel function and $\gamma > 0$ determines how the samples are transformed into a high-dimensional search space. As a result, data in higher dimensional space were linearly separated into two classes by the maximal-margin hyperplane, which provides the maximum distance between the two

classes by being located centrally between the marginal boundaries of each data class. The margin is defined as the distance between two marginal boundaries (2γ) where the support vectors are located.

The most critical step in constructing a well-generalized SVM classifier is to find the optimal parameters of the kernel function. In the case of non-linear SVM, parameters that should be considered include the complexity parameter (*C*), which searches for a balance between misclassification and simplicity, gamma (γ), which determines the extent to which one training sample affects the model, and epsilon (ε), which designates the exponent value (we note that a value for the linear kernel is 1).

To find the optimal parameters, a two-level grid search was performed using AutoWeka, which is a software program developed in-house. Initially, the global search was conducted by the systemic adjustment of exponential n values in the form of 2^n for C and γ parameters using a step size of 2. To obtain good performance, a more refined local grid search was performed on the regions from the global search using a step size of 0.25. The RMSE value was used for measurement of the predictive performance. Finally, SVM models were constructed by John Platt's Sequential Minimal Optimization (SMO) algorithm of the Weka software package version 3.7.11 (Witten et al., 2011) using the optimal parameters obtained from the local grid search. The optimal parameters for SVM analysis are shown in Table S4.

Table S4. Optimal parameters for the SVM models

**Model**	**Level of search**	**Complexity (C)**	**Gamma (γ)**	**Epsilon (ε)**	**$RMSE_{tr}$**	**$RMSE_{cv}$**
I_NI	global	19	-11	0.001[a]	0.3668	0.3678
I_NI	local[b]	21	-9.5	0.001[a]	0.3304	0.3474
S_NS	global	19	-1	0.001[a]	0.0000	0.0000
S_NS	local[b]	21	-0.75	0.001[a]	0.0000	0.0000

[a] Default ε value of 0.001 was used.

[b] Parameters of the local search were used for the construction of the SVM models.

References

Che, D., Liu, Q., Rasheed, K., & Tao, X. (2011). Decision Tree and Ensemble Learning Algorithms with Their Applications in Bioinformatics. In Software Tools and Algorithms for Biological Systems (Vol 696, pp. 191-199). H. R. Arabnia and Q.-N. Tran, New York, USA: Springer.

Nantasenamat, C., Li, H., Mandi, P., Worachartcheewan, A., Monnor, T., Isarankura-Na-Ayudhya, C., & Prachayasittikul, V. (2013a). Exploring the chemical space of aromatase inhibitors. *Molecular Diversity*, *17*(4), 661-677. DOI: 10.1007/s11030-013-9462-x.

Nantasenamat, C., Worachartcheewan, A., Prachayasittikul, S., Isarankura-Na-Ayudhya, C., & Prachayasittikul, V. (2013b). QSAR modeling of aromatase inhibitory activity of 1-substituted 1,2,3-triazole analogs of letrozole. *European Journal of Medicinal Chemistry*, *69*, 99-114. DOI: 10.1016/j.ejmech.2013.08.015.

Patil, T. R., & Sherekar, S. S. (2013). Performance Analysis of Naive Bayes and J48 Classification Algorithm for Data Classification. *International Journal of Computer Science and Applications*, *6*(2), 256-260.

Sharma, T. C., & Jain, M. (2013). WEKA Approach for Comparative Study of Classification Algorithm. *International Journal of Advanced Research in Computer and Communication Engineering*, *2*(4), 1925-1931.

Sutariya, V., Groshev, A., Sadana, P., Bhatia, D., & Pathak, Y. (2013). Artificial Neural Network in Drug Delivery and Pharmaceutical Research. *The Open Bioinformatics Journal*, *7*(Suppl-1,M5), 49-62. DOI: 10.2174/1875036201307010049.

Tarca, A. L., Carey, V. J., Chen, X. W., Romero, R., & Drăghici, S. (2007). Machine learning and its applications to biology. *PLoS computational biology*, *3*(6). DOI: 10.1371/journal.pcbi.0030116v.

Vapnik, V. N. (1998). *Statistical Learning Theory*. New York, USA: Wiley.

Vapnik, V. N. (2000). *The Nature of Statostical Learning Theory*. New York, USA: Springer.

Witten, I. H., Frank, E., & Hall, M. A. (2011). *Data Mining: Practical Machine Learning Tools and Techniques*. 3rd ed., San Francisco, CA, USA: Morgan Kaufmann.

Yang, Z. R. (2004). Biological applications of support vector machines. *Briefings in bioinformatics*, *5*(4), 328-338. DOI: 10.1093/bib/5.4.328.

Zhou, B., Ha, M.&Wang, C. (2010). An improved algorithm of unbalanced data SVM. *Advances in Intelligent and Soft Computing*, *78*, 549-555.
